# Detection of *Giardia* and helminths in Western Europe at local K9 (canine) sites (DOGWALKS Study)

**DOI:** 10.1186/s13071-022-05440-2

**Published:** 2022-09-03

**Authors:** Jason Drake, Sarah Sweet, Kingsley Baxendale, Evan Hegarty, Stephanie Horr, Hanne Friis, Troy Goddu, William G. Ryan, Georg von Samson-Himmelstjerna

**Affiliations:** 1 Elanco Animal Health, 2500 Innovation Way, Greenfield, IN 46140 USA; 2 IDEXX Laboratories, Inc., 1 IDEXX Dr, Westbrook, ME 04092 USA; 3 Elanco Animal Health, Bartley Wood Business Park, Hook, RG27 9XA UK; 4 IDEXX Europe B.V, Scorpius 60, Hoofddorp, The Netherlands; 5 Ryan Mitchell Associates LLC, Westfield, NJ 07090 USA; 6 Institute for Parasitology and Tropical Veterinary Medicine, Freie Universität Berlin, Berlin, Germany

**Keywords:** Ascarid, Centrifugal flotation, Coproantigen, Dog, Europe, *Giardia*, Hookworm, Whipworm

## Abstract

**Background:**

Intestinal parasite contamination from infected dogs can place other dogs and humans at risk. A study was initiated to estimate the prevalence of canine intestinal parasitism by collecting fecal samples in cities across Western Europe.

**Methods:**

Fresh fecal samples were collected from 2469 dogs visiting 164 parks in 33 cities across 12 countries. Each owner responded to a questionnaire focusing on their dog’s signalment and recent anthelmintic treatment history. The collected samples were examined for hookworms, whipworms, ascarids and *Giardia* using a coproantigen diagnostic immunoassay and microscopy following centrifugal flotation.

**Results:**

Nematodes or *Giardia* were detected in at least one sample from 100% of cities and in 93.3% of parks. Nematodes were detected in 57% of parks. Overall, 22.8% of dogs tested positive for an intestinal parasite, with *Giardia* being the most commonly identified parasites (17.3% of dogs, 83.5% of parks). For nematode infection, 7.6% of all dogs tested positive, with 9.9% of dogs aged < 1 year infected, 7.7% of those aged 1–3 years, 7.3% of those aged 4–6 years and 6.6% of those aged ≥ 7 years. Among the nematodes detected, ascarids were the most prevalent (3.6% of dogs, parks, 28.7% of parks), being most common in dogs aged < 1 year but also present in older dogs, including those aged ≥ 7 years. Hookworms and whipworms were detected in 3.2% and 2.3% of dogs of all ages, respectively, and in 37.2% and 17.7% of parks, respectively. A larger proportion of fecal samples tested positive with the coproantigen immunoassay than with centrifugal flotation. Positive test results for *Giardia* were sevenfold higher when both diagnostic tests were used than when centrifugal flotation alone was used, and there were 60% more positive test results for nematodes when both tests were used than when flotation alone was used. Overall, 77.2% of owners reported previous anthelmintic treatment, among whom at least 62.7% failed to follow recommended treatment frequency. Dogs receiving anthelmintic within the previous month had a lower percentage of nematode infection than those in which > 1 month had passed since the previous dose.

**Conclusions:**

The prevalence estimates of intestinal parasite infections in dogs reported here highlight the need for owner education concerning guidelines for regular testing and treatment, even in older dogs. Failure to adhere to guidelines can result in ongoing transmission of these infections, including those with zoonotic potential. Combining coproantigen immunoassay with centrifugal flotation for diagnostic testing and regular anthelmintic treatment are important measures for ensuring optimal intestinal parasite control.

**Graphical Abstract:**

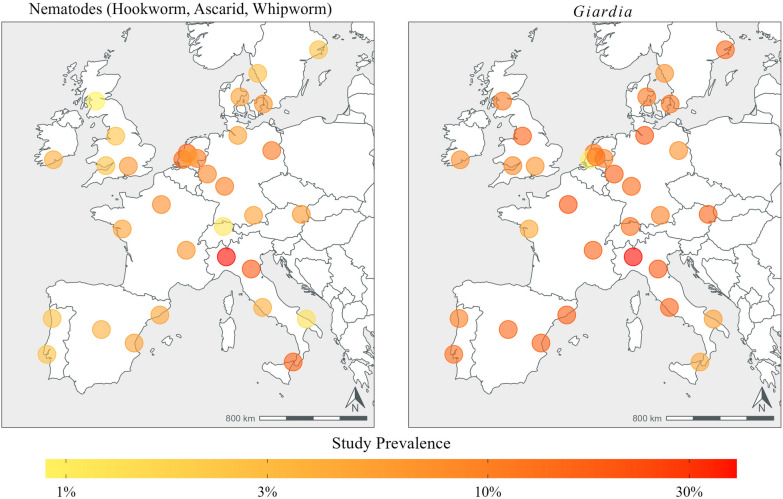

**Supplementary Information:**

The online version contains supplementary material available at 10.1186/s13071-022-05440-2.

## Background

Intestinal parasitism presents potential health risks to canine hosts, and environmental contamination from infected dogs, often subclinical, can place other dogs, other animals and humans at risk of disease. While shelter and stray dogs are at the greatest risk for parasite infection, reports from multiple European countries indicate that intestinal parasite infections are common in household dogs [1–9]. *Ancylostoma caninum*, *Trichuris vulpis* and *Toxocara canis* have been the most commonly identified nematode species in dogs, and infections with *Giardia intestinalis* (syn. *G. duodenalis* and *G. lamblia*), *Cystoisospora* spp. and tapeworms are common. An understanding of the risk of canine intestinal parasite infection, the diagnosis of infection and effective control measures are therefore important considerations from a One Health perspective.

Insights into the risk of canine intestinal parasitism can be provided by prevalence surveys. Most such reporting is based on findings from traditional methods for detecting infections, including fecal smears, standing gravitational passive flotation and centrifugal flotation. Smears and passive flotation have been shown to be insensitive for the detection of intestinal parasites, and while centrifugal flotation is more reliable than smears and passive flotation, this technique can sometimes fail to detect infections, such as those in the prepatent period or in cases where parasite egg excretion is very low, intermittent or not uniformly dispersed within the feces [10–12].

Unlike fecal flotation methods, recently introduced coproantigen immunoassays detect infections during the prepatent period, prior to eggs being shed, thereby increasing the sensitivity of testing for common intestinal nematode species as well as *Giardia* [11–14]. The commercial availability of immunoassays provides an opportunity to generate further insights into the prevalence of canine intestinal parasitism by identifying infections missed by flotation methods.

In this report, we describe the first European multi-country study to estimate the prevalence of canine intestinal parasite infections, focusing on pet dogs being exercised by their owners in local parks, as opposed to shelter dogs or dogs presented for veterinary care, using two testing methods: a zinc sulfate centrifugal flotation test and a validated coproantigen assay. The primary study objective was to estimate the proportion of dogs infected with intestinal nematodes (roundworms, hookworms, whipworms) and *Giardia* by collecting fresh fecal samples in cities and areas in Western Europe where dogs are commonly exercised. Secondary objectives were to determine the benefit of combined testing with the coproantigen immunoassay and fecal flotation for the detection of parasites, and to investigate the relationship between owner-reported anthelmintic medication and the presence of intestinal parasites.

## Methods

### Fecal sample collection locations

A collection site was determined as an area in a city (including the surrounding region) where people commonly walk or exercise their dogs, whether on or off the lead. Trained investigators collected fresh fecal samples from 2469 dogs visiting 164 sites in 33 cities across 12 countries (Table 1). In order to cover a range of socioeconomic areas in each city, investigators selected five separate collection sites in parks or other appropriate areas in each city, allowing for geographic and socioeconomic diversity and safety considerations.

**Table 1:** Cities selected for collection of samples

Country (abbreviation)	Cities
Austria (AT)	Vienna
Denmark (DK)	Aarhus, Copenhagen
France (FR)	Lyon, Nantes, Paris
Germany (DE)	Berlin, Cologne, Frankfurt, Hamburg, Munich
Ireland (IE)	Cork
Italy (IT)	Bari, Bologna, Messina, Pavia, Rome
Netherlands (NE)	Amsterdam, Arnhem/Nijmegen, Rotterdam, Utrecht
Portugal (PT)	Lisbon, Porto
Spain (ES)	Barcelona, Madrid, Valencia
Sweden (SE)	Gothenburg, Stockholm
Switzerland (CH)	Zurich
United Kingdom (UK)	Cardiff, Glasgow, Manchester, Reading

### Selected dogs for sampling and communication with owners

All dogs from which fecal samples were collected were owned by (or under the care of) the site visitors whose participation in the study was voluntary. For inclusion in the study, the dog’s owner, or someone in the household of the dog’s owner, must have been present with the dog, agreed for the sample to be collected and verbally responded to the study questionnaire on the dog’s signalment and recent anthelmintic treatment (Additional file 1: Table S1). Only a single sample was collected from any one dog, and just one dog was sampled from households with multiple dogs. Dogs were included regardless of age, breed, sex, stool quality or clinical status. Dogs in the care of professional dog walkers or individuals who were not familiar with the dog’s home conditions were excluded. No study samples were collected from dogs belonging to any employees of the sponsoring companies, nor from those belonging to any investigator, any veterinary clinic staff member or any academic staff member known to an investigator. The questionnaire was completed on site.

### Fecal sample collections and testing

Investigators included veterinarians and other staff employed by Elanco Animal Health (Greenfield, IN, USA) or IDEXX Laboratories, Inc. (Westbrook, ME, USA), local practicing veterinarians, university veterinarians and associated staff and veterinary students. All were trained as study investigators to collect or to supervise collections. No more than 20 dogs were to be sampled at any one site. Each sample was immediately placed into a hard-plastic container onto which a pre-printed sticker was placed. Collected sample containers were placed into a specimen bag and sent in an insulated box to a commercial laboratory (IDEXX GmbH, Kornwestheim, Germany; IDEXX Laboratories Ltd., Wetherby UK). Each fecal sample was tested for primary nematodes (hookworms, whipworms and ascarids) and *Giardia* using a coproantigen immunoassay (Fecal Dx® and *Giardia* Test; IDEXX Laboratories, Inc.) and centrifugal flotation using a zinc sulfate solution (specific gravity: 1.24–1.27).

### Analysis of results

The percentage of positive samples for each parasite or parasite category was calculated and the confidence interval (CI) estimated using the binomial exact method. Comparison of the percentages of positive samples according to anthelmintic use was done by age-weighting each group to control for the confounding effect of age. Comparisons were made between samples from dogs whose owners indicated no anthelmintic use, those whose owners indicated anthelmintic use within the last month and those whose owners indicated a longer time lapse since last anthelmintic dosage. Within each of these three groups, the number of samples positive for primary nematodes were weighted so that their age distributions were identical to that of the entire study population. Differences between these groups were considered by comparing confidence intervals estimated from the age-weighted percentages. All analyses were done using R version 4.1.0 [15].

### Protocol deviations

Protocol deviations included a change of cities for collections in Italy, Spain and Portugal due to availability of investigators. Samples from one site in Barcelona were lost in transit. In France, COVID-19 restrictions mandated collection be limited to three of the five originally selected cities. In five cities, there were changes in originally selected sites due to insufficient numbers of dogs to allow collection of the minimum number of samples or elimination of the original site from the location. There were 11 cases that were classified as ineligible because either a sample or completed questionnaire was not received, and in five cases mislabeling was identified and corrected.

## Results

### Demographics

**Fig. 1:**
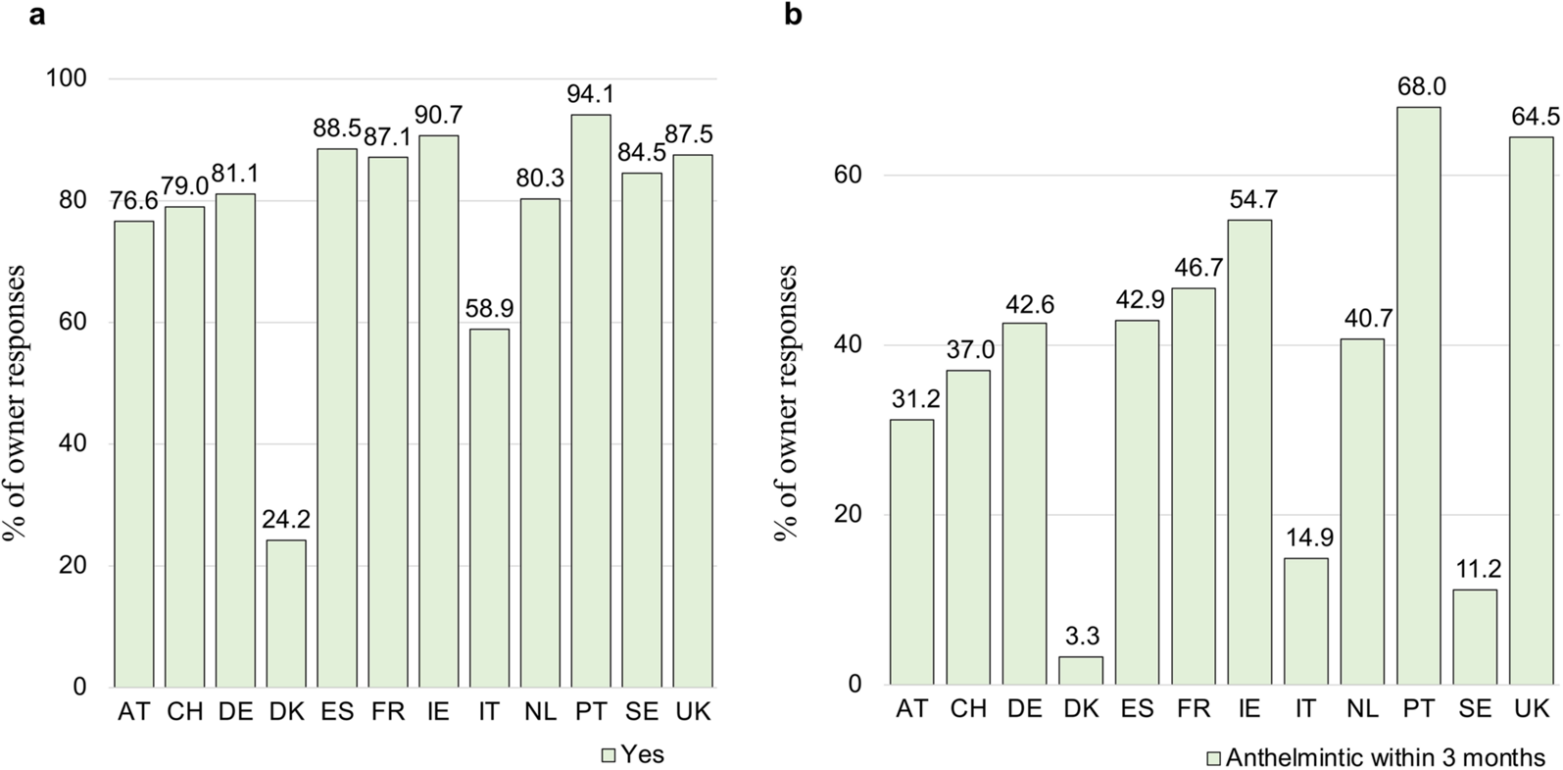
The percentage of each country’s dog owner responses to the question “Does the dog receive heartworm, lungworm, or intestinal worm medication?” **a** Answer “Yes”; **b** Answer to treatment within 1 month and within 1–3 months (i.e. within 3 months of sampling). See Table 1 for country abbreviation

From March through September 2021, fresh fecal samples were collected and examined from 2469 dogs whose owners completed the study questionnaire, across 164 parks in 12 countries. Of the 2469 dogs sampled, 72.3% weighed > 10 kg, 12.3% were aged < 1 year and 57.6% were aged > 3 years (1.1% had age unknown) and there were similar proportions of female and male dogs although the proportion of intact male dogs was higher than that of intact female dogs (51.6% vs 41.4%). In terms of breed category, the most common description (40.5%) was “Other”, indicating that most dogs were of mixed breed; of the remaining categories, the five most commonly described breeds were Labrador Retriever (6.1%), Golden Retriever (3.6%), Jack Russell Terrier (2.7%), Beagle (2.3%) and Dachshund (2.1%). In response to the question on previous anthelmintic treatment of their dog, 77.2% of owners (*n* = 1906) responded “Yes,” ranging from 24.2% (*n* = 37) among Danish respondents to 94.1% (*n* = 144) among Portuguese respondents (Fig. 1a), and 16.0% responded “No”; 6.8% of owners did not know whether or not a treatment had been administered. The median percentage of positive responses across countries was 83%. Of the 77.2% of owners who responded “Yes” to previous anthelmintic treatment of their dog, 16.5% stated that treatment had occurred within the previous month, 33.2% within 1–3 months, 20.4% within 4–6 months, 10.9% within 7–12 months and 13.9% not within the previous 12 months (5.1% did not recall the timing). A history of intestinal worm infection in their dog was reported by 17.6% of owners. Of these owners, 92% responded “Yes” to having administered anthelmintic to their dog, while only 80% of dog owners without this history responded “Yes,” as did 59% of owners of dogs with unknown history of infection. Of the owners responding “Yes” to having administered anthelmintics, those reporting administering treatment within the previous 3 months made up the highest proportion of those in the UK and Portugal and lowest proportion of those in Italy, Denmark and Sweden (Fig. 1b).

### Fecal test results

Fecal tests using centrifugal flotation and the coproantigen immunoassay detected at least one positive sample for primary nematodes or *Giardia* in 100% of cities in the study and in 93.3% of parks (Table 2). In addition, at least one sample that was positive for a primary nematode was collected in 57% of parks. Test results also showed that 22.8% of samples were positive for the primary nematodes or *Giardia*. The most commonly identified parasites were *Giardia*, the most common nematode diagnosis was ascarid, followed by hookworm and whipworm. Of the 55 samples positive for ascarid eggs using centrifugal flotation, 52 were identified as *Toxocara* spp. and four as *Toxascaris leonina* (1 dog was positive for both ascarid species). For the 47 samples that tested positive for hookworm, 40 were identified by flotation as *Uncinaria stenocephala* and two as *Ancylostoma* spp. (both from France), while for five samples the hookworm species was not differentiated. Larvae of the lungworm *Angiostrongylus vasorum* were identified in three samples (2 from Denmark; 1 from Switzerland). Spurious findings included *Eimeria* spp. (2.8% of tested samples), strongyle and tapeworm eggs of herbivores (0.5% and < 0.1%, respectively) and *Hymenolepis diminuta* (0.1%) (a full summary of findings is included in Additional file 2: Table S2). The most common intestinal parasite coinfection was ascarid with *Giardia* in 33 dogs (1.3%), and the most common mixed nematode infection was ascarid with whipworm in 16 dogs (0.6%).

**Table 2:** Descriptive statistics of dogs and parks with at ≥ 1 sample positive for nematodes and other parasites

Fecal tests^a^	Dogs (*N* = 2469)	Parks (*N* = 164)
Dogs positive for any parasitic species^b^	589 (23.9)	153 (93.3)
	95% CI: 22.2–25.6	
Dogs positive for primary nematodes^c^ or *Giardia*	563 (22.8)	151 (93.2)
	95% CI: 21.2–24.5	
Dogs positive for *Giardia*	428 (17.3)	137 (83.5)
	95% CI: 15.9–18.9	
Dogs positive for primary nematodes^c^	188 (7.6)	94 (57.3)
	95% CI: 6.6–8.7	
Ascarid	89 (3.6)	47 (28.7)
	95% CI: 2.9‒4.4	
Hookworm	78 (3.2)	61 (37.2)
	95% CI: 2.5‒3.9	
Whipworm	56 (2.3)	29 (17.7)
	95% CI: 1.7‒2.9	
*Cystoisospora* spp.	26 (1.1)	20 (12.2)
	95% CI: 0.7‒1.5	
Capillarids	10 (0.4)	9 (5.5)
	95% CI: 0.2‒0.7	
Apicomplexa	6 (0.2)	5 (3.0)
	95% CI: 0.1‒0.5	
Lungworm	4 (0.2)	4 (2.4)
	95% CI: 0.0‒0.4	
*Strongyloides stercoralis*	1 (< 0.1)	1 (0.6)
	95% CI: 0.0‒0.2	

Descriptive statistics presented in table are the number, the percentage (in parentheses) and the confidence interval (CI) (as appropriate)

^a^Combined results of coproantigen immunoassay and centrifugal flotation

^b^Includes all diagnosed parasites and spurious observations

^c^Primary nematodes: hookworm, whipworm, ascarid

**Fig. 2:**
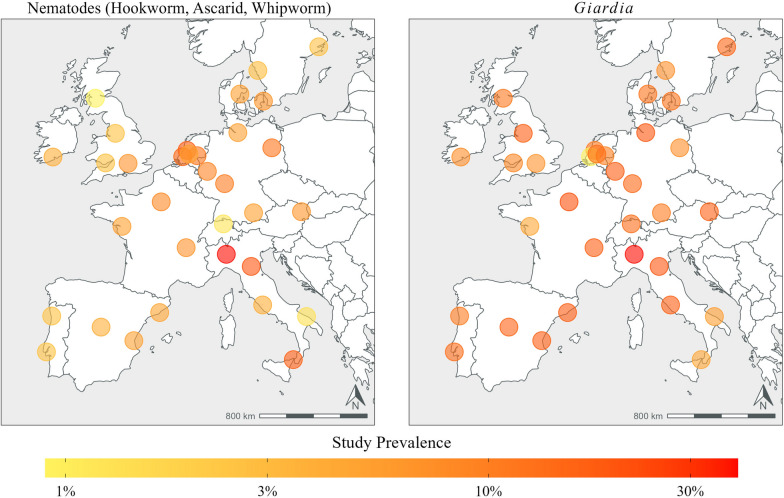
Heat map showing prevalence of canine intestinal parasites in sampled cities

Notable among the between-country findings is that the two countries with the highest prevalence of nematodes, including the highest prevalence of ascarid findings, were Italy and the Netherlands, each with > 10% of samples positive. Countries with > 5% of nematode-positive tests were Austria, Denmark, France, Germany and Ireland (Table 3; Fig. 2).

**Table 3:** Descriptive statistics of dogs positive for intestinal parasite infections in each country from which fresh fecal samples were collected

Country (number of samples)	*Giardia*/Nematodes	Nematode^a^	Hookworm	Whipworm	Ascarid	*Giardia*
Austria (*n *= 77)	20 (26.0)	5 (6.5)	2 (2.6)	0 (0.0)	4 (5.2)	17 (22.1)
	95% CI: 16.6‒37.2	95% CI: 2.1‒14.5	95% CI: 0.3‒9.1	95% CI: 0.0‒4.7	95% CI: 1.4‒12.8	95% CI: 13.4‒33.0
Denmark (*n* = 153)	32 (20.9)	9 (5.9)	6 (3.9)	2 (1.3)	2 (1.3)	24 (15.7)
	95% CI: 14.8‒28.2	95% CI: 2.7‒10.9	95% CI: 1.5‒8.3	95% CI: 0.2‒4.6	95% CI: 0.2‒4.6	95% CI: 10.3‒22.4
France (*n* = 225)	56 (24.9)	15 (6.7)	9 (4.0)	4 (1.8)	2 (0.9)	42 (18.7)
	95% CI: 19.4‒31.1	95% CI: 3.8‒10.8	95% CI: 1.8‒7.5	95% CI: 0.5‒4.5	95% CI: 0.1‒3.2	95% CI: 13.8‒24.4
Germany (*n* = 371)	91 (24.5)	31 (8.4)	18 (4.9)	9 (2.4)	10 (2.7)	68 (18.3)
	95% CI: 20.2‒29.2	95% CI: 5.7‒11.7	95% CI: 2.9‒7.6	95% CI: 1.1‒4.6	95% CI: 1.3‒4.9	95% CI: 14.5‒22.6
Ireland (*n* = 75)	11 (14.7)	5 (6.7)	3 (4.0)	0 (0.0)	3 (4.0)	6 (8.0)
	95% CI: 7.6‒24.7	95% CI: 2.2‒14.9	95% CI: 0.8‒11.2	95% CI: 0.0‒4.8	95% CI: 0.8‒11.2	95% CI: 3.0‒16.6
Italy (*n* = 375)	120 (32.0)	61 (16.3)	18 (4.8)	34 (9.1)	27 (7.2)	88 (23.5)
	95% CI: 27.3‒37.0	95% CI: 12.7‒20.4	95% CI: 2.9‒7.5	95% CI: 6.4‒12.4	95% CI: 4.8‒10.3	95% CI: 19.3‒28.1
Netherlands (*n* = 295)	66 (22.4)	36 (12.2)	5 (1.7)	0 (0.0)	32 (10.8)	32 (10.8)
	95% CI: 17.7‒27.6	95% CI: 8.7‒16.5	95% CI: 0.6‒3.9	95% CI: 0.0‒1.2	95% CI: 7.5‒15.0	95% CI: 7.5‒15.0
Portugal (*n* = 153)	27 (17.6)	4 (2.6)	2 (1.3)	1 (0.7)	1 (0.7)	24 (15.7)
	95% CI: 12.0‒24.6	95% CI: 0.7‒6.6	95% CI: 0.2‒4.6	95% CI: 0.0‒3.6	95% CI: 0.0‒3.6	95% CI: 10.3‒22.4
Spain (*n* = 217)	53 (24.4)	10 (4.6)	6 (2.8)	3 (1.4)	5 (2.3)	49 (22.6)
	95% CI: 18.9‒30.7	95% CI: 2.2‒8.3	95% CI: 1.0‒5.9	95% CI: 0.3‒4.0	95% CI: 0.8‒5.3	95% CI: 17.2‒28.7
Sweden (*n* = 143)	23 (16.1)	4 (2.8)	2 (1.4)	1 (0.7)	3 (2.1)	21 (14.7)
	95% CI: 10.5‒23.1	95% CI: 0.8‒7.0	95% CI: 0.2‒5.0	95% CI: 0.0‒3.8	95% CI: 0.4‒6.0	95% CI: 9.3‒21.6
Switzerland (*n* = 81)	15 (18.5)	1 (1.2)	1 (1.2)	0 (0.0)	0 (0.0)	14 (17.3)
	95% CI: 10.8‒28.7	95% CI: 0.0‒6.7	95% CI: 0.0‒6.7	95% CI: 0.0‒4.5	95% CI: 0.0‒4.5	95% CI: 9.8‒27.3
UK (*n* = 304)	49 (16.1)	7 (2.3)	6 (2.0)	2 (0.7)	0 (0.0)	43 (14.1)
	95% CI: 12.2‒20.7	95% CI: 0.9‒4.7	95% CI: 0.7‒4.2	95% CI: 0.1‒2.4	95% CI: 0.0‒1.2	95% CI: 10.4‒18.6

Descriptive statistics presented in table are the number, the percentage (in parentheses) and the confidence interval (CI)

Table shows combined results of coproantigen immunoassay and centrifugal flotation tests

^a^≥ 1 of hookworm, whipworm or ascarid detected

**Fig. 3:**
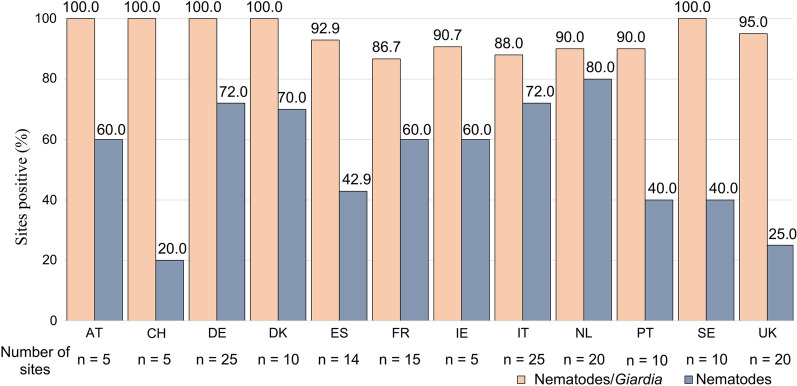
Percentage of parks in each country in which at least one sample was positive for canine intestinal parasites–*Giardia* and nematodes (hookworm, whipworm and ascarid)

*Giardia* was detected from at least one site in every country from which samples were collected, and at least one sample was positive for nematodes in at least 20% of parks in all countries. Ascarids were detected in samples from 70% of parks in Netherlands and from approximately 30–40% of parks in Austria, Germany, Ireland, Italy, Spain and Sweden (Fig. 3; Table 4).

**Table 4:** Number of sites positive for individual parasites

Country (number of sites)	Hookworm	Whipworm	Ascarid	*Giardia*
Austria (*n* = 5)	2 (40.0%)	0 (0.0%)	2 (40.0%)	5 (100.0%)
Denmark (*n* = 10)	6 (60.0%)	2 (20.0%)	2 (20.0%)	10 (100.0%)
France (*n* = 15)	6 (40.0%)	1 (6.7%)	2 (13.3%)	13 (86.7%)
Germany (*n* = 25)	15 (60.0%)	8 (32.0%)	8 (32.0%)	24 (96.0%)
Ireland (*n* = 5)	2 (40.0%)	0 (0.0%)	2 (40.0%)	2 (40.0%)
Italy (*n* = 25)	10 (40.0%)	12 (48.0%)	9 (36.0%)	21 (84.0%)
Netherlands (*n* = 20)	5 (25.0%)	0 (0.0%)	14 (70.0%)	9 (45.0%)
Portugal (*n* = 10)	2 (20.0%)	1 (10.0%)	1 (10.0%)	9 (90.0%)
Spain (*n* = 14)	6 (42.9%)	2 (14.3%)	4 (28.6%)	13 (92.9%)
Sweden (*n* = 10)	2 (20.0%)	1 (10.0%)	3 (30.0%)	9 (90.0%)
Switzerland (*n *= 5)	1 (20.0%)	0 (0.0%)	0 (0.0%)	5 (100.0%)
UK (*n* = 20)	4 (20.0%)	2 (10.0%)	0 (0.0%)	17 (85.0%)

**Fig. 4:**
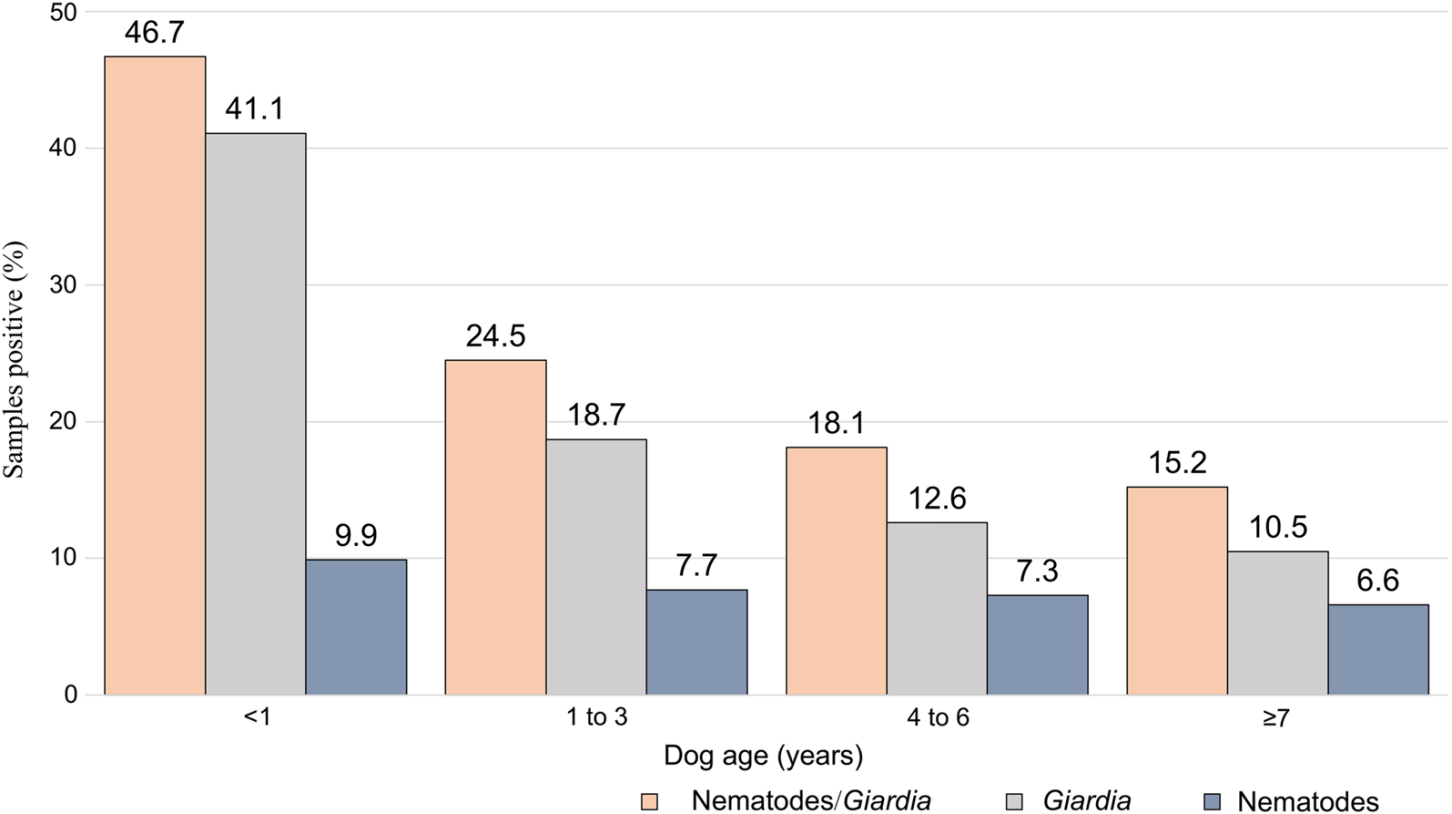
Percentage of dogs by age group with fecal samples positive for intestinal parasites

**Fig. 5:**
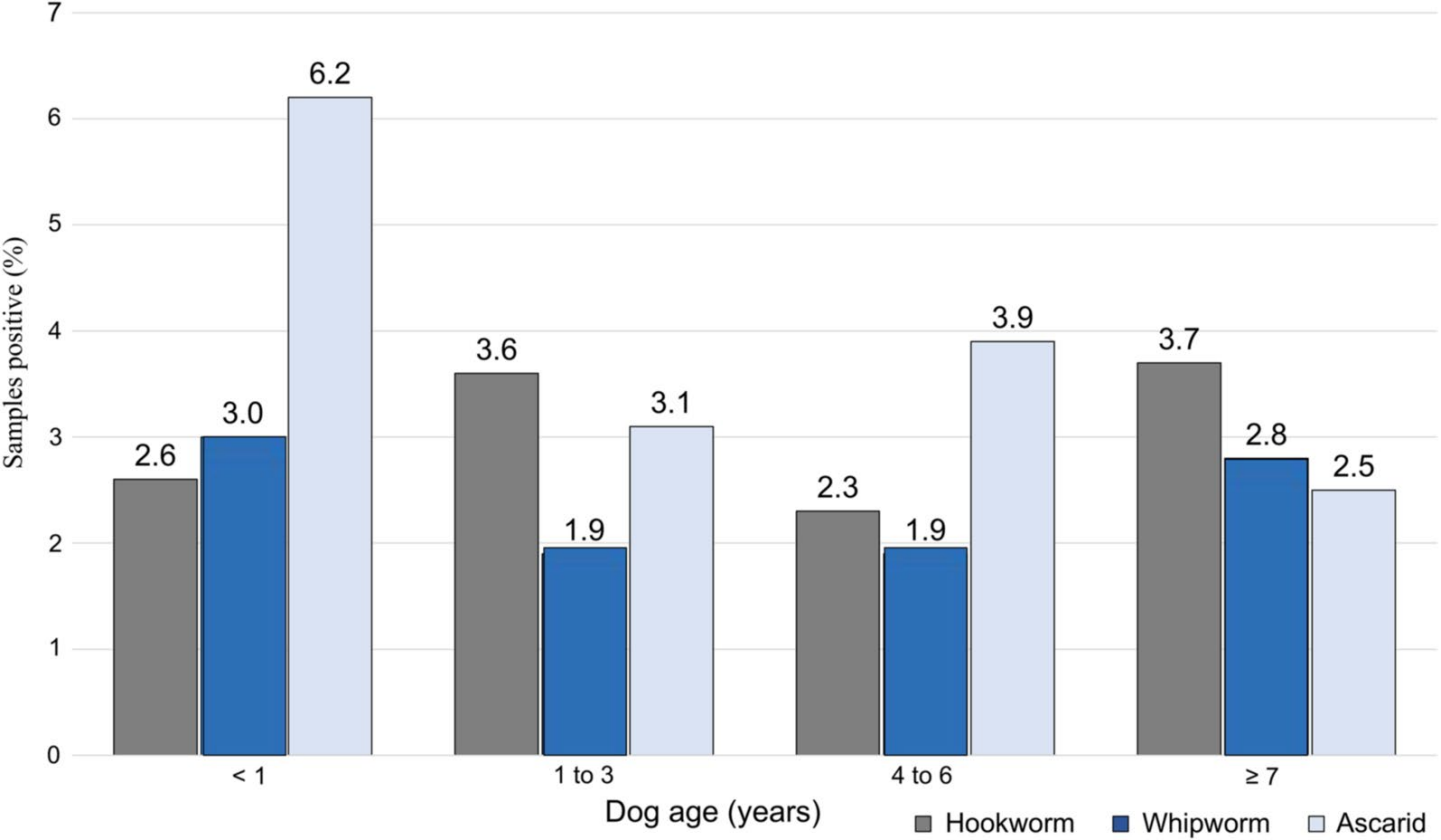
Percentage of dogs by age group with fecal samples positive for hookworms, whipworms and ascarids

Infection with intestinal parasites was most prevalent in dogs aged < 1 year and declined with increasing age (Table 5; Fig. 4). Ascarids were the most commonly found intestinal nematode in the young age group (< 1 year), but eggs were detected across all age groups, including from dogs that were at least 7 years old (Fig. 5). This age trend in positive samples was driven mostly by positive tests for *Giardia* and ascarids.

**Table 5:** Descriptive statistics of fecal samples positive for hookworms, whipworms and ascarids according to age group of dogs

Age (no. of samples)	*Giardia*/Nematodes	Nematode^a^	Hookworm	Whipworm	Ascarid	*Giardia*
< 1 year (*n* = 304)	142 (46.7)	30 (9.9)	8 (2.6)	9 (3.0)	19 (6.2)	125 (41.1)
	95% CI: 41.0‒52.5	95% CI: 6.8‒13.8	95% CI: 1.1‒5.1	95% CI: 1.4‒5.5	95% CI: 3.8‒9.6	95% CI: 35.5‒46.9
1–3 years (*n* = 718)	176 (24.5)	55 (7.7)	26 (3.6)	14 (1.9)	22 (3.1)	134 (18.7)
	95% CI: 21.4‒27.8	95% CI: 5.8‒9.9	95% CI: 2.4‒5.3	95% CI: 1.1‒3.2	95% CI: 1.9‒4.6	95% CI: 15.9‒21.7
4–6 years (*n* = 696)	126 (18.1)	51 (7.3)	16 (2.3)	13 (1.9)	27 (3.9)	88 (12.6)
	95% CI: 15.3‒21.2	95% CI: 5.5‒9.5	95% CI: 1.3‒3.7	95% CI: 1.0‒3.2	95% CI: 2.6‒5.6	95% CI: 10.3‒15.3
≥ 7 years (*n* = 725)	110 (15.2)	48 (6.6)	27 (3.7)	20 (2.8)	18 (2.5)	76 (10.5)
	95% CI: 12.6‒18.0	95% CI: 4.9‒8.7	95% CI: 2.5‒5.4	95% CI: 1.7‒4.2	95% CI: 1.5‒3.9	95% CI: 8.3‒12.9
Age not known (*n* = 26)	9 (34.6)	4 (15.4)	1 (3.8)	0 (0.0)	3 (11.5)	5 (19.2)
	95% CI: 17.2‒55.7	95% CI: 4.4‒34.9	95% CI: 0.1‒19.6	95% CI: 0.0‒13.2	95% CI: 2.4‒30.2	95% CI: 6.6‒39.4

Descriptive statistics presented in table are the number, the percentage (in parentheses) and the confidence interval (CI)

^a^≥ 1 of hookworm, whipworm or ascarid detected

**Fig. 6:**
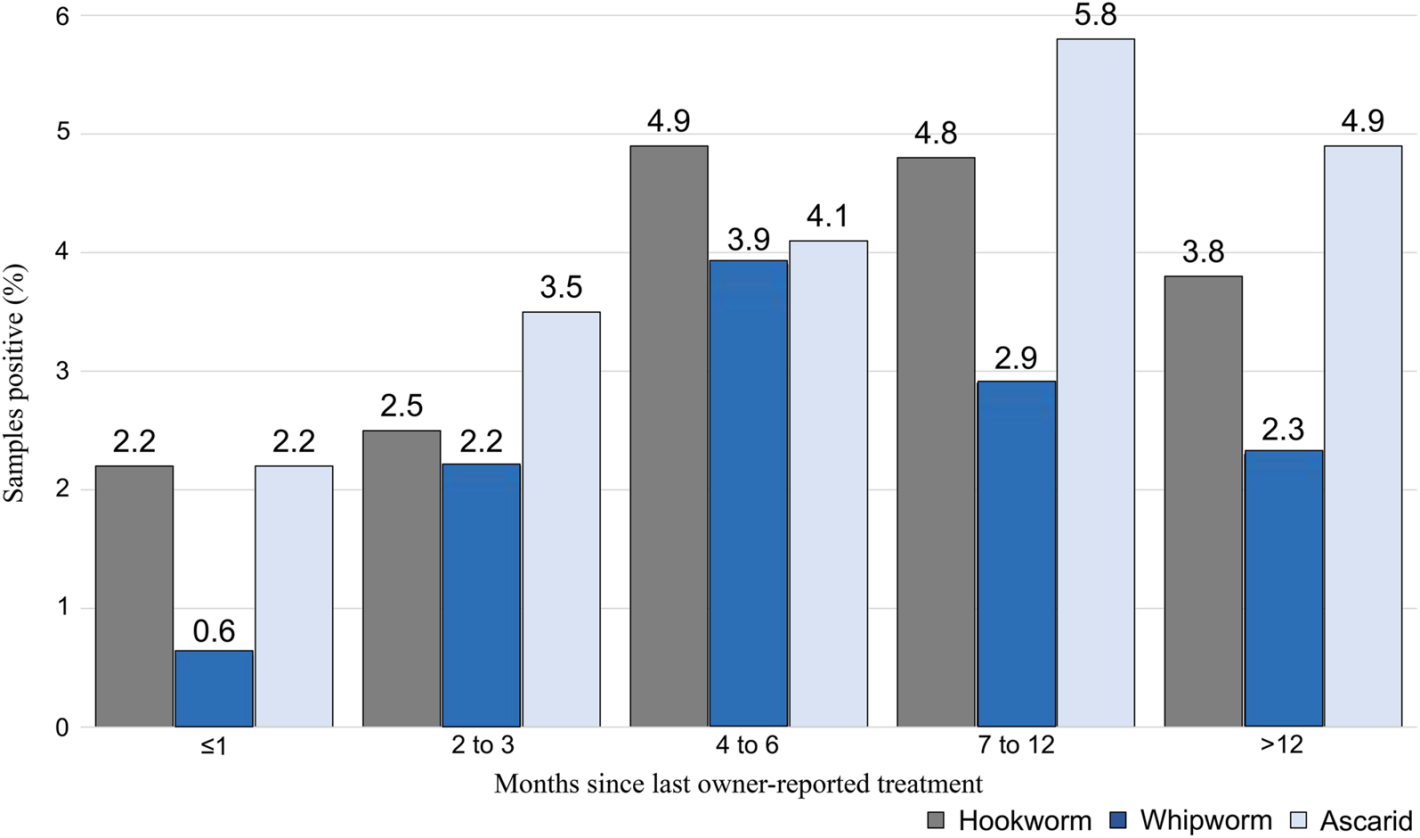
Percentage of dogs with fecal samples positive for nematode parasites according to owner-reported timing of the last anthelmintic administration (“Don’t remember” infection percentages positive not included)

The estimated percentage of fecal samples positive for nematode infection collected from dogs reported by their owners to have received anthelmintic medication was 7.8% (95% CI: 6.6–9.1%), and the percentage of fecal samples positive for nematode infection collected from dogs not receiving such medication was 5.3% (95% CI: 3.3‒8.0%) (Table 6; Fig. 6). For *Giardia*, 17.7% (95% CI: 16.0‒19.5%) of dogs that had been reported to have been administered anthelmintics tested positive and 15.2% (95% CI: 11.8‒19.2%) of those reported not to be receiving anthelmintic were positive. Confidence intervals based on the weighted percentage of nematode-positive tests showed that dogs whose owners reported anthelmintic use in the previous month had a lower percentage of nematode infection than did dogs with owners who reported a period of > 1 month since the last anthelmintic dose. Dogs whose owners reported no anthelmintic use had a weighted percentage of nematode-positive tests between those of the other two groups.

**Table 6:** Detected proportion of intestinal parasites by owner-reported time of last anthelmintic administration

Medication frequency (number of samples)	*Giardia*/nematode	Nematode^a^	Hookworm	Whipworm	Ascarid	*Giardia*
≤ 1 month (*n* = 315)	78 (24.8)	15 (4.8)	7 (2.2)	2 (0.6)	7 (2.2)	64 (20.3)
	95% CI: 20.1‒29.9	95% CI: 2.7‒7.7	95% CI: 0.9‒4.5	95% CI: 0.1‒2.3	95% CI: 0.9‒4.5	95% CI: 16.0‒25.2
> 1 to 3 months (*n* = 633)	150 (23.7)	42 (6.6)	16 (2.5)	14 (2.2)	22 (3.5)	121 (19.1)
	95% CI: 20.4‒27.2	95% CI: 4.8‒8.9	95% CI: 1.5‒4.1	95% CI: 1.2‒3.7	95% CI: 2.2‒5.2	95% CI: 16.1‒22.4
4 to 6 months (*n* = 389)	116 (29.8)	44 (11.3)	19 (4.9)	15 (3.9)	16 (4.1)	85 (21.9)
	95% CI: 25.3‒34.6	95% CI: 8.3‒14.9	95% CI: 3.0‒7.5	95% CI: 2.2‒6.3	95% CI: 2.4‒6.6	95% CI: 17.8‒26.3
7 to 12 months (*n* = 208)	49 (23.6)	22 (10.6)	10 (4.8)	6 (2.9)	12 (5.8)	33 (15.9)
	95% CI: 18.0 ‒ 29.9	95% CI: 6.7‒15.6	95% CI: 2.3‒8.7	95% CI: 1.1‒6.2	95% CI: 3.0‒9.9	95% CI: 11.2‒21.6
> 12 months (*n* = 264)	43 (16.3)	22 (8.3)	10 (3.8)	6 (2.3)	13 (4.9)	27 (10.2)
	95% CI: 12.0‒21.3	95% CI: 5.3‒12.3	95% CI: 1.8‒6.9	95% CI: 0.8‒4.9	95% CI: 2.6‒8.3	95% CI: 6.8‒14.5
Don’t remember (*n* = 97)	11 (11.3)	3 (3.1)	2 (2.1)	0 (0.0)	1 (1.0)	8 (8.2)
	95% CI: 5.8‒19.4	95% CI: 0.6‒8.8	95% CI: 0.3‒7.3	95% CI: 0.0‒3.7	95% CI: 0.0‒5.6	95% CI: 3.6‒15.6

Values in table are presented as the number, the percentage (in parentheses) and the confidence interval (CI)

^a^Primary nematode is ≥ 1 of hookworm, whipworm or ascarid detected

### Results by methodology

**Fig. 7:**
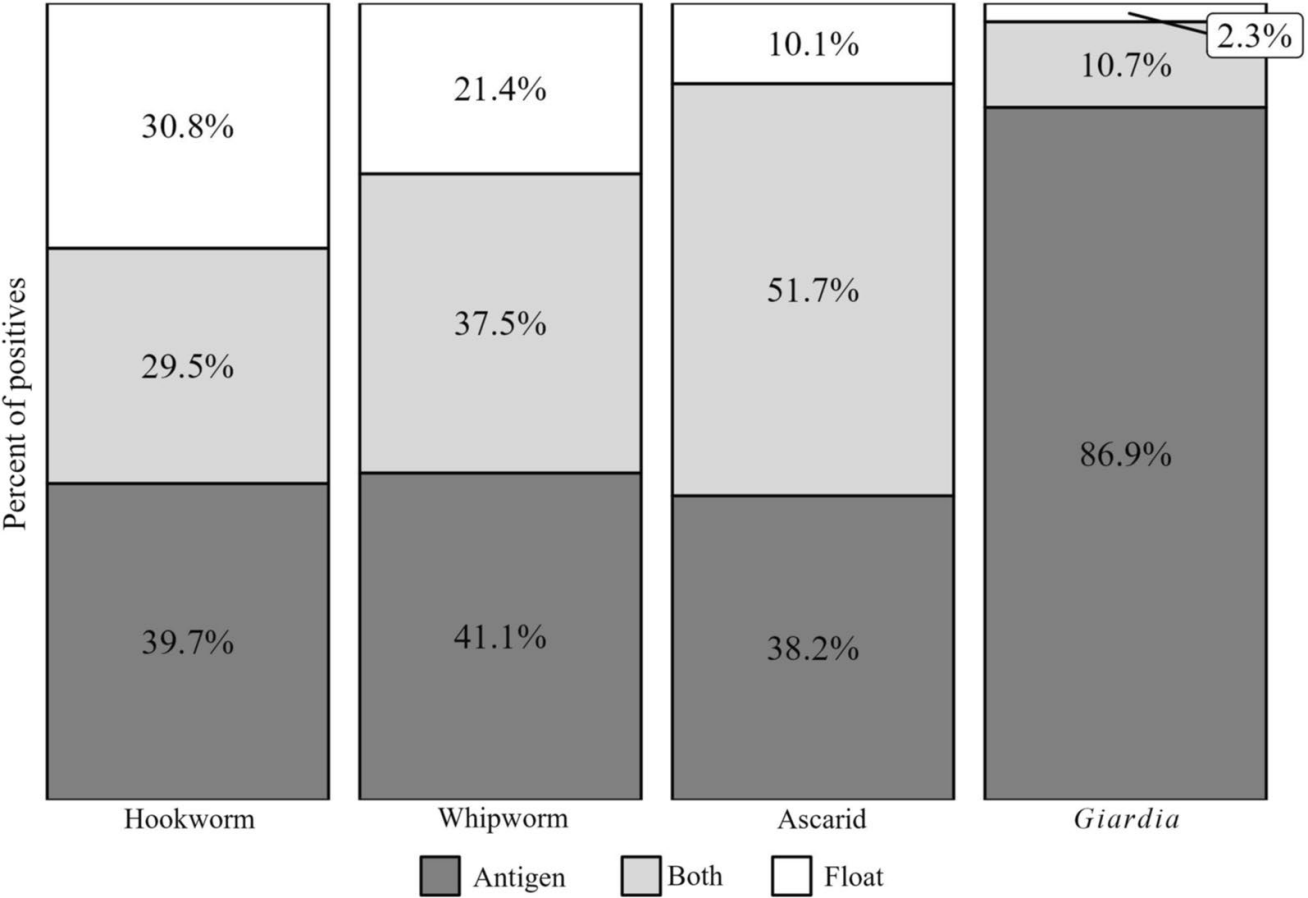
For each primary nematode and *Giardia*, the percentage of samples positive for that parasite identified by coproantigen immunoassay only, centrifugal flotation only, and by both methods

A larger proportion of test results were positive for the coproantigen immunoassay compared with the centrifugal flotation for the primary nematodes and *Giardia*. For the primary nematodes, 154 of the 188 positive samples (81.9%) were identified using the coproantigen immunoassay and 118 (62.8%) were identified using centrifugal flotation. The respective numbers for the 428 samples positive for *Giardia* were 418 (97.7% of positive samples) and 56 (13.1%). Thirty-one samples that tested positive for hookworm by coproantigen immunoassay were negative by centrifugal flotation, while 24 samples that tested positive for hookworm by centrifugal flotation were negative on the coproantigen immunoassay; the test results agreed for 23 of the 78 hookworm-positive samples (29.5%) (Fig. 7). For whipworms, 44 positive samples (78.6% of the total positive samples) were detected using the coproantigen immunoassay and 33 (58.9%) were detected by centrifugal flotation. For ascarids, the coproantigen immunoassay detected 80 positive samples (89.9% of the total that were positive) while centrifugal flotation detected 55 positive samples (61.8%).

## Discussion

This report describes the first multi-city, multi-country investigation carried out in Europe to estimate the prevalence of intestinal parasitism by examining fresh fecal samples collected from pet dogs while exercising in the company of their owners. Samples testing positive for intestinal parasitism were identified in every city in every country and from > 90% of parks in those cities, suggesting that any dog exercised outside of the home can be at risk of infection. The methodology used in the present study was similar to that of a 2019 study conducted across the USA (the DOGPARCS study) in which fresh fecal samples were collected from over 3000 dogs [16]. Similar to the results in the USA study, in the present study *Giardia* was the most prevalent intestinal parasite detected, and the overall prevalence of the primary nematodes was comparable (in that study 8.8% of dogs and 49.7% of parks, compared with 7.6% of dogs and 57.3% of parks in this study). However, ascarid prevalence was much lower in the DOGPARCS study than in the present study (0.6% vs 3.6%), and hookworm prevalence much higher (7.1% [predominantly *A. caninum*] vs 3.2% [predominantly *U. stenocephala*]).

A number of limitations to the present study must be acknowledged. First, collections were completed from each park within a short period of just 1 or 2 days, so that a limited number of dogs and parks were sampled, resulting in a potential underestimate of the presence of intestinal parasites. Second, the number of cities sampled in each country was too low to allow extrapolation of findings to the country as a whole, especially for Ireland, Austria and Switzerland where collections were completed from just a single city. Third, samples were collected country by country spanning spring through late summer; consequently, the seasonal variations in prevalence are not captured by the data, potentially influencing between-country comparisons. Importantly, by collecting samples through the spring–summer period, seasonal fluctuations of infections with ascarids (highest seasonal prevalence suggested as December and January) and whipworms (highest prevalence in January and February) would have been missed, potentially leading to an underestimation of the prevalence of these nematodes specifically [17]. Fourth, the study was designed to provide insight into the prevalence of the primary nematodes and *Giardia*. The results do not provide any indication of the prevalence of cestodes and lungworms because the methodologies used were not sensitive for their detection (although *A. vasorum* and *C. vulpis* were detected in a number of samples). Fifth, samples positive for *Toxocara* spp. by flotation were not further analyzed to differentiate between *T. canis* and *T. cati*, leaving potential *T. cati* eggs resulting from coprophagia unnoted [18]. However, *T*. *cati* and *T*. *canis* are host specific, meaning a positive *Toxocara* spp. antigen result in this study is highly unlikely to be an active *T*. *cati* infection [18]. Finally, while the results demonstrate that pet dogs can be a source of environmental parasite contamination, those from which samples were collected were accompanied by their owners, approximately three quarters of whom report providing some anthelmintic treatment. As free-roaming and shelter dogs are more likely to have intestinal parasite burdens than those included in our study, our results may underestimate the prevalence of canine intestinal parasite infection rate and the resulting health risk arising from the wider dog population [2, 6, 9]. Allowing for these limitations, the results do provide a sound basis for guidance on the presence of intestinal parasitism of pet dogs in Europe with *Giardia* and nematodes.

The high *Giardia* prevalence found in this study aligns with that reported in other studies completed throughout Europe and beyond (reviewed by Bouzid et al. [19]) that often identify it as the most common canine intestinal parasite infection [16, 19, 20]. In those studies, the authors reported a higher proportion of younger than older dogs were positive for *Giardia*. In our study, combining the coproantigen immunoassay and centrifugal flotation technique detected more than sevenfold the number of positive tests for *Giardia* than flotation alone (16.9% coproantigen/2.3% centrifugal flotation/17.3% combined), consistent with other reports showing that flotation is unreliable for the detection of canine infection with this parasite [4, 19, 21]. That unreliability may be attributed to both the intermittent excretion and fragility of cysts, which can degenerate between fecal sample collection and examination at the laboratory, and to potential disruption caused by the osmotic pressure of a concentrated zinc sulfate solution [16].

The combined use of the coproantigen assay and flotation would be a contributing factor to the relatively high proportion of dogs that tested positive for *Giardia* in the present study compared to the reported overall prevalence of up to 7% in European dogs and cats [22]. Additionally, over half of the dogs who tested positive for *Giardia* infection in this study were aged < 3 years. Nonetheless, the 12.0% of positive *Giardia* findings from flotation tests in our study is substantially higher than the ESCCAP (European Scientific Counsel for Companion Animal Parasites) estimate (3–7% in dogs and cats, significantly higher in young animals), although it is broadly consistent with other reports of a prevalence ranging from 9% to 16% in dogs in Belgium and Germany, respectively, and much lower than the 30–41.0% found in other studies in Europe [4, 8, 23–27]. While the data do not provide a sound basis to conclude that a park visit increases the risk of canine infection with *Giardia*, it may be possible that the gathering of dogs in parks provides greater potential for exposure, particularly if the environment encourages congregation around water sources.

Dogs reportedly receiving an anthelmintic within 1 month prior to sample collection were significantly less often positive for nematodes than those treated > 1 month previously, after accounting for age differences in these groups. Unexpectedly, dogs not receiving anthelmintics had a slightly lower percentage of positive results for nematodes than dogs reported to be receiving anthelmintics, although this difference was not significant. While we accounted for differences in age, other lifestyle factors (e.g. home environment, frequency of exposure to infection sources) could have impacted the positive findings in these groups. One explanation may be that as time from administration increased, owners confused other medications (e.g. flea/tick treatment) with anthelmintic. Additionally, having been advised during the interview that the study objective was to detect intestinal parasites in their dog, some owners may have felt social pressure to respond positively to the question on the use of anthelmintic. Thus, across countries a surprisingly high country-level median percentage of respondents (83%) indicated that their dog had been treated. However, the results suggest that > 60% of owners had not treated their dog within the previous 3 months, consistent with earlier surveys showing that there are widespread failures in adhering to recommendations of ESCCAP regarding the frequency of anthelmintic treatment [28–32]. Those recommendations state that dogs with outdoor access and contact with other dogs or parks should be tested or treated at least 4 times per year to provide effective control of ascarids and other intestinal helminth parasites.

Owner failure to adhere to expert recommendations is a likely contributing factor to the relatively high prevalence of ascarids that were found in all age groups, including older dogs. Ascarids continue to be of concern, not least because of their zoonotic potential. Adult female *T. canis* are capable of producing up to 85,000 eggs per day; these eggs are resistant to common disinfectants, are largely refractory to extreme environmental conditions and can survive for years to infect other dogs and non-canine hosts, including humans [33]. That prolificity, together with the environmental hardiness of eggs, lack of susceptibility of inhibited stages to most anthelmintic drug treatments, transplacental transmission so that puppies are born with infections and dog-owner failure to follow testing and treatment guidelines are all factors underlying the continuing high prevalence of this zoonotic parasite. Following infection, the migratory larvae have been diagnosed as the cause of a range of human neurological, ophthalmologic, pulmonary and cutaneous symptoms [34]. The prevalence of positive tests in dogs aged between 4 and 6 years and ≥ 7 years serves as a reminder of the need for continued testing and treatment of dogs of all ages, including older dogs that may act as important reservoirs of infection, even if the infection is not clinically apparent (Fig. 4). Other reports, including a recent review, have shown that the prevalence of *Toxocara* spp. infections has remained worryingly high over many decades and, with few exceptions, our study results are consistent with these earlier findings [34–38].

In the present study, we did not establish any clear trends between the proportions of nematode-positive tests and owners’ recollection of timing of the latest anthelmintic use. However, it is worthy of mention that of the countries with results from at least 100 dogs and 10 sites, Portugal and the UK had the highest proportion of owners responding that they had treated their dog within the previous 3 months (68.0% and 64.5%, respectively), and the lowest percentage of ascarid eggs was detected in samples from these two countries (0.7% and 0.0%). In contrast, some countries with lower owner-reported anthelmintic administration within the previous 3 months, specifically Italy (14.9%) and the Netherlands (40.7%), had the highest prevalence of ascarid-positive tests (7.2% and 10.8%, respectively). Italy also had the highest proportion of positive tests for whipworm and was a close second to Germany for positive tests for hookworm (43.4% of dogs reportedly treated with anthelmintic within the previous 3 months).

Hookworms, which were most prevalent in Germany but detected in all countries, appeared to be mainly *U. stenocephala.* Only two samples, both from France, were positive for *A. caninum* which has zoonotic potential and a life-cycle favored by tropical and subtropical climates, as does its potentially zoonotic (but less pathogenic) relative *Ancylostoma braziliense.* The short life-cycle of *A. caninum* (prepatent period: 2–3 weeks), prolific egg-laying (25,000 eggs/adult per day) and travel/importation of infected pets provide the potential for a rapid increase in risk of infection. This was shown in the USA where between 2015 and 2018 there was a 47% increase in reported prevalence of hookworms [17]. Many European reports describe a high prevalence of Ancylostomatidae, relative to other canine intestinal parasites, without providing details on species [1–5]. It would be helpful if future studies differentiate hookworm species, now of growing importance with the worrying emergence of multi-drug resistant *A. caninum* [39, 40].

Whipworms were identified in samples from all countries except Austria, Ireland, Switzerland and the Netherlands. Sampling of only a single city in Austria, Ireland and Switzerland limited the ability to detect whipworms. The highest prevalence of *T. vulpis* was found in Italy, which also had the highest prevalence of primary nematodes and the lowest-but-one percentage of owners reporting use of anthelmintic (Denmark had the lowest such use, likely related to it being the only country in this study with a requirement for a positive test or clinical diagnosis before treatment can be dispensed [41]). Potentially a cause of severe illness and death, due to the intermittent egg excretion and the high density of the eggs, infections can often be missed by commonly used fecal flotation methods [42].

Combined used of the coproantigen immunoassay and centrifugal flotation technique detected an additional (approximately) 60% more positive results for nematodes than flotation alone (coproantigen: 6.2%; centrifugal flotation: 4.8%; combined: 7.6%), suggesting that there is a benefit from combining both tests for each sample. The higher proportion of positive tests from the coproantigen immunoassay could be related to the detection of prepatent infections that are missed by flotation. Samples testing positive on flotation but negative on immunoassay may be due to predation or coprophagia, as evidenced by findings of spurious parasites not infective to dogs, such as *Eimeria* spp. (2.8%) and strongyle eggs of herbivores (0.5%), or to the lack of sufficient antigen production from low-intensity infections [16].

Overall, the results show that intestinal parasite-infected dogs are visiting and potentially contaminating public parks, exposing other dogs to infection. While our protocol required the collection of samples from common canine exercise areas, there was no comparison made between dogs in other areas, such as those being walked on streets, or whether dogs were on or off the lead. Consequently, the results should not be seen as a problem exclusive to dogs being exercised in parks. What the results do show is that the cost of dog-owner failures to adhere to ESCCAP recommendations is the increased potential for their dog to be infected and to spread potentially zoonotic nematode parasites. That cost highlights the need for owner and veterinary education on the benefit of adherence to ESCCAP recommendations: (i) to pick up and carefully dispose of dog feces (which will also help to limit dissemination of potentially zoonotic *Giardia*); (ii) to test and treat “indoor” dogs for intestinal nematodes at least once or twice per year; and (iii) for the dog-owner population visiting parks with their dogs, such as those included in the present study, to administer an effective anthelmintic at least 4 and up to 12 times per year [15].

## Conclusions

The prevalence estimates of intestinal nematode infections found in dogs exercised in parks in Western Europe, including dogs in older age groups, highlight the need for owner education regarding ESCCAP recommendations for regular testing and treatment. The study findings indicate that failure to adhere to those recommendations can result in the ongoing transmission of intestinal parasite infections, including those with zoonotic potential, risking the health of other dogs, humans and other animals. The combined use of coproantigen assays and centrifugal flotation for testing and the administration of comprehensive anthelmintic treatment at an appropriate frequency are critical measures for ensuring optimal parasite control.

## Supplementary Information

**Additional file 1: Table S1. **DOGPARCS Study owner questionnaire.

**Additional file 2: Table S2. **Complete list of findings from centrifugal flotation identifications.

## Data Availability

Data supporting the conclusions of this article are included within the article and its additional file. Raw data from this study are available for review upon reasonable request to Elanco Animal Health or IDEXX.
