# Supplementary material for: Detection of Giardia and helminths in Western Europe at local K9 (canine) sites (DOGWALKS Study)
Source: Parasit Vectors. 2022 Sep 3;15:311. doi: 10.1186/s13071-022-05440-2 (PMC9440314; doi:10.1186/s13071-022-05440-2)
Supplement: Supplementary file 1 — Additional file 1: Table S1. DOGPARCS Study owner questionnaire. [file 13071_2022_5440_MOESM1_ESM.docx]

**Additional file 1: Table S1DOGPARCS Study Owner Questionnaire**

| 1. Investigator's Name | | | (First, Last) | | | | | | | | | | | | | | | | | |
| --- | --- | --- | --- | --- | --- | --- | --- | --- | --- | --- | --- | --- | --- | --- | --- | --- | --- | --- | --- | --- |
| 1. Animal ID | | |  | | | | | | | | | | | | | | | |  |  |
| 1. Breed of Dog | | | | (Indicate breed. If mixed- or cross-bred, enter MIXED. If unknown, enter UNK) | | | | | | | | | | | | | | |  |  |
| 1. Sex of Dog   (Select One) | | □ Intact Female | | | | □ Spayed Female | | | | □ Intact  Male | | | | | □ Neutered Male | | | | | |
| 1. Age   (Select One) | □ ≤ 1 year | | | | □ 1-3 years | | | □ 4-6 years | | | | □ ≥ 7 years | | | | | □ Unknown | | |  |
| 1. Weight   (Select One) | □ 1-5 kg | | | | □ 6-10 kg | | | □ 11-25 kg | | | | □ 26-45 kg | | | | | □ > 45 kg | | |  |
| 7. Does dog receive heartworm, lungworm, or intestinal worm medication? (Select One) | | | | | | | | | □ Yes | | | | □ No | | | □ Unknown | |  |  |  |
| If yes, when was the dog last treated? (Select One) | | | | | | | □ Within the last month (30 days)  □ Within the last 1 - 3 months  □ Within the last 4 - 6 months  □ Within the last 7 - 12 months  □ Over 12 months ago  □ Don't remember/Don't know | | | | | | | | | | | | | |
| 1. Has the dog ever had intestinal worms? (Select One) | | | | | | | | | | | □Yes | | | □ No | | □ Unknown | | | | |
