# Supplementary material for: Detection of Giardia and helminths in Western Europe at local K9 (canine) sites (DOGWALKS Study)
Source: Parasit Vectors. 2022 Sep 3;15:311. doi: 10.1186/s13071-022-05440-2 (PMC9440314; doi:10.1186/s13071-022-05440-2)
Supplement: Supplementary file 2 — Additional file 2: Table S2. Complete list of findings from centrifugal flotation identifications. [file 13071_2022_5440_MOESM2_ESM.docx]

**Additional file 2: Table S2**. Complete list of findings from centrifugal flotation identifications

| **Species** | **Positive (%)** |
| --- | --- |
| *Cyniclomyces guttulatus* yeasts | 154 (6.2%) |
| *Eimeria* spp. | 69 (2.8%) |
| *Giardia* | 56 (2.3%) |
| *Toxocara* spp. | 52 (2.1%) |
| *Uncinaria stenocephala* | 40 (1.6%) |
| *Trichuris* spp. | 33 (1.3%) |
| Mite eggs | 31 (1.3%) |
| Free living nematodes | 22 (0.9%) |
| *Isospora burrowsi ohioensis* | 22 (0.9%) |
| Strongyle eggs of herbivores | 12 (0.5%) |
| *Capillaria* spp. | 10 (0.4%) |
| *Monocystis* spp. | 6 (0.2%) |
| *Sarcocystis* spp. | 6 (0.2%) |
| Hookworm spp. | 5 (0.2%) |
| *Myocoptes musculinus* | 5 (0.2%) |
| *Toxascaris leonina* | 4 (0.2%) |
| *Angiostrongylus vasorum* | 3 (0.1%) |
| *Hymenolepis diminuta* | 3 (0.1%) |
| *Cystoisospora* spp. | 3 (0.1%) |
| *Ancylostoma* spp. | 2 (<0.1%) |
| Ascarid eggs of birds | 2 (<0.1%) |
| *Isospora canis* | 2 (<0.1%) |
| Tapeworm eggs of herbivores | 2 (<0.1%) |
| *Adelina* spp. | 1 (<0.1%) |
| *Capillaria* sp. eggs of birds | 1 (<0.1%) |
| *Cheyletiella* spp. | 1 (<0.1%) |
| *Crenosoma vulpis* | 1 (<0.1%) |
| Fly maggots | 1 (<0.1%) |
| *Strongyloides* spp. | 1 (<0.1%) |
| *Trichuris* eggs of herbivores | 1 (<0.1%) |
